# Supplementary figures and images for: Genetic characterization of Angiostrongylus larvae and their intermediate host, Achatina fulica, in Thailand
Source: PLoS One. 2019 Sep 27;14(9):e0223257. doi: 10.1371/journal.pone.0223257 (PMC6764694; doi:10.1371/journal.pone.0223257)

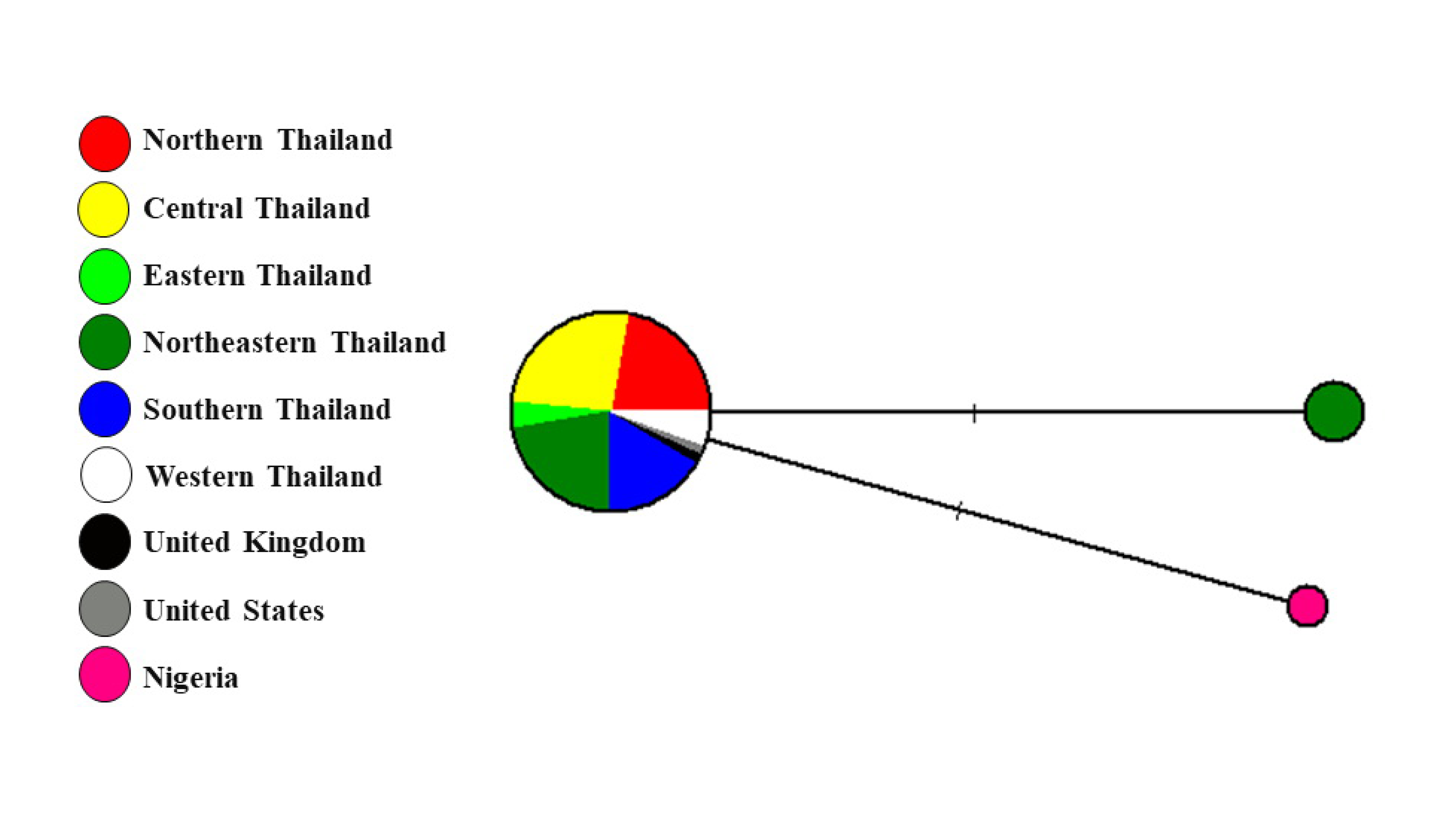

Supplement: S1 Fig — Each haplotype is represented by a circle. Sizes of circles are relative to number of individuals sharing specific haplotype. (TIF) [file pone.0223257.s001.tif]
