# Supplementary material for: Genetic characterization of Angiostrongylus larvae and their intermediate host, Achatina fulica, in Thailand
Source: PLoS One. 2019 Sep 27;14(9):e0223257. doi: 10.1371/journal.pone.0223257 (PMC6764694; doi:10.1371/journal.pone.0223257)
Supplement: S1 Table — (PDF) [file pone.0223257.s002.pdf]

S1 Table. Number of *Achatina fulica* used for artificial digestion method collected across Thailand.

Locality	No. of snail for artificial digestion method	Result	No. of larvae
**North**			
Uttaradit	2	N	
Chiang Rai	21	P (*A. malaysiensis*)	1,269
Chiang Mai	3	N	
Nan	22	N	
Phrae	1	P (*A. malaysiensis*)	13
	29	N	
**Central**			
Phitsanulok	3	N	
Phetchabun	5	N	
Bangkok	30	N	
Lop Buri	1	N	
Phra Nakhon Si Ayutthaya	27	N	
Nakhon Sawan	31	N	
**East**			
Rayong	12	N	
**Northeast**			
Nakhon Ratchasima	2	N	
Buri Ram	15	N	
Maha Sarakham	17	N	
Chaiyaphum	30	P (*A. cantonensis*)	313
	9	N	
Udon Thani	6	N	
Nakhon Phanom	3	N	
**South**			
Pattani	30	N	
Chumphon	11	N	
Surat Thani	21	N	
**West**			
Prachuap Khiri Khan	12	N	

P, Positive; N, negative.
