# Supplementary material for: Genetic characterization of Angiostrongylus larvae and their intermediate host, Achatina fulica, in Thailand
Source: PLoS One. 2019 Sep 27;14(9):e0223257. doi: 10.1371/journal.pone.0223257 (PMC6764694; doi:10.1371/journal.pone.0223257)
Supplement: Attachment — Submitted filename: Response to Reviewers.docx [file pone.0223257.s010.docx]

PONE-D-19-18473
Genetic characterization of Angiostrongylus larvae and their natural intermediate host, Achatina fulica, in Thailand
PLOS ONE

Journal Requirements:

1. When submitting your revision, we need you to address these additional requirements.

Please ensure that your manuscript meets PLOS ONE's style requirements, including those for file naming. The PLOS ONE style templates can be found at
http://www.journals.plos.org/plosone/s/file?id=wjVg/PLOSOne_formatting_sample_main_body.pdf and http://www.journals.plos.org/plosone/s/file?id=ba62/PLOSOne_formatting_sample_title_authors_affiliations.pdf

*We do follow the requirements of PLOS ONE style.*

2. In your Methods section, please provide additional information regarding the permits you obtained for the work. Please ensure you have included the full name of the authority that approved the field site access and, if no permits were required, a brief statement explaining why.

*No specific permits were required for snail collection in the specific area. We have added this sentence “No specific permission was required for sampling snail in public locations.” in the method 🡪 Collection of Achatina fulica*

Additional Editor Comments:

The MS brings informative results of the distribution of Angiostrongylus and the snail intermediate host in Thailand. However, improvement on the text is necessary. The methodology should be written in a more concise manner. The map presented as a supplementary material could be a figure with data of snails/haplotypes and Angiostrongylus/haplotypes. Also in the discussion, the correlation of haplotype and human infection (if possible) would be an interesting and informative point in this MS.

*Thank for the positive comment to our manuscript. We do follow your suggestion as;*

- *We have deleted several sentences from the method section “extraction of genomic DNA”*
- *We have included the map of Thailand with distribution of A. fulica and Angiostrongylus haplotypes as Fig 1 in the text.*
- *We have rewrite some parts of the discussions.*

Reviewers' comments:

Reviewer's Responses to Questions

**Comments to the Author**

1. Is the manuscript technically sound, and do the data support the conclusions?

The manuscript must describe a technically sound piece of scientific research with data that supports the conclusions. Experiments must have been conducted rigorously, with appropriate controls, replication, and sample sizes. The conclusions must be drawn appropriately based on the data presented. 

Reviewer #1: Yes

Reviewer #2: Yes

*Thank for comments*

2. Has the statistical analysis been performed appropriately and rigorously? 

Reviewer #1: I Don't Know
Reviewer #2: N/A

*-*

3. Have the authors made all data underlying the findings in their manuscript fully available?

The [PLOS Data policy](http://www.plosone.org/static/policies.action#sharing) requires authors to make all data underlying the findings described in their manuscript fully available without restriction, with rare exception (please refer to the Data Availability Statement in the manuscript PDF file). The data should be provided as part of the manuscript or its supporting information, or deposited to a public repository. For example, in addition to summary statistics, the data points behind means, medians and variance measures should be available. If there are restrictions on publicly sharing data—e.g. participant privacy or use of data from a third party—those must be specified.

Reviewer #1: No
Reviewer #2: Yes

*Thank for comments*

4. Is the manuscript presented in an intelligible fashion and written in standard English?

PLOS ONE does not copyedit accepted manuscripts, so the language in submitted articles must be clear, correct, and unambiguous. Any typographical or grammatical errors should be corrected at revision, so please note any specific errors here.

Reviewer #1: Yes
Reviewer #2: Yes

*Thank for comments*

5. Review Comments to the Author

Please use the space provided to explain your answers to the questions above. You may also include additional comments for the author, including concerns about dual publication, research ethics, or publication ethics. (Please upload your review as an attachment if it exceeds 20,000 characters)

Reviewer #1: PONE-D-19-18473

This is a very nice set of data, with genetic studies of Angiostrongylus and its intermediate host in a wide an important endemic area.

*Thank you for your comments.*

The title: what do authors mean by “natural”? I can think of two meanings: “that can be found naturally infected” and “well-adapted”. I suggest to remove this word from the title since it does not add any fundamental information besides the main objective.
*We have deleted the word “natural” from the title so the title have changed as reviewer suggested.*

*“Genetic* *characterization of Angiostrongylus larvae and their intermediate host, Achatina fulica, in Thailand”*

Line 73: As commented above, I see no reason to include “in nature”.
*We have deleted the word “in nature” as reviewer suggested.*

Line 84- Sure that genetic studies are fundamental, but the linkage between this study and “disease control” or specifically angiostrongyliasis control is not clear. Perhaps authors may re-write or clarify.
“Genetic characterization is important for the identification of parasitic pathogens, as well as to control the spreading of disease.” This sentence is described the general for study genetics.

We think that study on genetic of parasite is important for indirect control the disease. Because we use molecular identification for diagnosis or confirm species of parasites, it will be useful for select the appropriate measure control methods for specific parasites. In addition, genetic study on detection of drug resistant gene in parasites may help for select the appropriate drug for control parasite.

Line 100- “…an invasive land snail..” is not needed here as this was just addressed in the previous section.
*We have deleted the phrase “an invasive land snail” as reviewer suggested.*

Tables- formatting shall be revised according to PLoS One instructions.

*We have changed the Table format as reviewer suggested*

Figures- They also need improvement for better quality.

*We have changed the resolution of all Figures presented in the text for better quality as reviewer suggested.*

Figure 1- the map of Thailand showing places where collection was performed. A much nicer presentation would be the indication of collection places with dots corresponding to exact geographic coordinates (geo-referencing). This shall be the informative mode of presenting geographic occurrence data, since just painting the geographic area of province is less informative.

*We have added the Figure 1 in the text as reviewer suggested.*

Results- one very important information is the distribution of individual larvae counts. I strongly suggest authors to provide this information in a suppplementary file or to add it in manuscript and explore it. It gives a very nice opportunity to discuss the degree of parasite-host adaptation. It also enriches the contribution of this nice work.

*We have added the data on the infection of A. cantonensis larvae in the host in the supplementary data as “S1 Table”*

Reviewer #2:

Comment to MS: MS. No. PONE-D-19-18473 Genetic characterization of Angiostrongylus larvae and their natural intermediate host, Achatina fulica, in Thailand
This study revealed genetic diversity of Angiostrongylus larvae and Achatina fulica, natural intermediate host. New findings of A. fulica haplotypes were found (AF1 and AF2) based on sequencing of the COI gene. Two new additional cytb haplotypes of A. cantonensis (AC19 and AC20) were also identified in this study. Scientific sound is good. Manuscript is well written. This is useful for more understanding of system study to explore the epidemiology of Angiostrongylus spp. and Achatina fulica intermediate host.

*Thank you for positive comments on our manuscript.*

Specific comment:
For ensure phylogenetic analysis, in addition to using the maximum-likelihood (ML) method in Phylogenetic analysis of Figures 1, 2 and 3, the authors should also use other methods such as (Bayesian analyses (BI) maximum-parsimony (MP) and neighbour-joining (NJ) analyses implemented as reported by as Ref 9: Rodpai R, Intapan PM, Thanchomnang T, Sanpool O, Sadaow L, Laymanivong S, et al. Angiostrongylus cantonensis and A. malaysiensis Broadly Overlap in Thailand, Lao PDR, Cambodia and Myanmar: A Molecular Survey of Larvae in Land Snails. PLoS One. 2016;11(8): e0161128. https://doi.org/10.1371/journal.pone.016112.

*We have analyzed the BI, MP and NJ methods and have changed Figs 2-6 by adding the support values in the phylogenetic tree as reviewer suggested.*

6. PLOS authors have the option to publish the peer review history of their article ([what does this mean?](https://journals.plos.org/plosone/s/editorial-and-peer-review-process#loc-peer-review-history)). If published, this will include your full peer review and any attached files.
If you choose “no”, your identity will remain anonymous but your review may still be made public.
**Do you want your identity to be public for this peer review?** For information about this choice, including consent withdrawal, please see our [Privacy Policy](https://www.plos.org/privacy-policy).
Reviewer #1: Yes: Carlos Graeff-Teixeira
Reviewer #2: No


[NOTE: If reviewer comments were submitted as an attachment file, they will be attached to this email and accessible via the submission site. Please log into your account, locate the manuscript record, and check for the action link "View Attachments". If this link does not appear, there are no attachment files to be viewed.]
While revising your submission, please upload your figure files to the Preflight Analysis and Conversion Engine (PACE) digital diagnostic tool,<https://pacev2.apexcovantage.com/>. PACE helps ensure that figures meet PLOS requirements. To use PACE, you must first register as a user. Registration is free. Then, login and navigate to the UPLOAD tab, where you will find detailed instructions on how to use the tool. If you encounter any issues or have any questions when using PACE, please email us at [figures@plos.org](mailto:figures@plos.org). Please note that Supporting Information files do not need this step.
